# CUL2 overexpression driven by CUL2/E2F1/miR-424 regulatory loop promotes HPV16 E7 induced cervical carcinogenesis

**DOI:** 10.18632/oncotarget.9127

**Published:** 2016-05-02

**Authors:** Junfen Xu, Yifeng Fang, Xinyu Wang, Fenfen Wang, Qifang Tian, Ying Li, Xing Xie, Xiaodong Cheng, Weiguo Lu

**Affiliations:** ^1^ Department of Gynecologic Oncology, Women's Hospital, School of Medicine, Zhejiang University, Hangzhou 310006, China; ^2^ The Second Department of General Surgery, Sir Run Run Shaw Hospital, School of Medicine, Zhejiang University, Hangzhou 310016, China

**Keywords:** CUL2, HPV16 E7, miR-424, E2F1, cervical cancer

## Abstract

It has been shown that HPV16 E7, but not other genotypes, can bind to scaffold protein CUL2 during inducing cervical carcinogenesis, but the expression level, associated regulating mechanism, and potential carcinogenicity of CUL2 itself is still unknown as yet. Here, we demonstrated that CUL2 was specifically overexpressed in HPV16 positive cervical cancer cells and tissues, and CUL2 expression was significantly increased along with the cervical lesion progression and positively correlated with HPV16 E7. CUL2 knockdown slowed the growth of xenograft tumors in mouse models. Importantly, CUL2 specifically bound to HPV16 E7, but not HPV18 E7. Moreover, CUL2 acted as a direct target of miR-424, and reversely suppressed miR-424; E2F transcription factor 1 (E2F1) suppressed miR-424 expression; CUL2 bound to E2F1 and promoted E2F1 expression. Our results indicate the existence of a regulatory loop among CUL2, E2F1, and miR-424 in HPV16 positive cervical cancer cells. Our results suggest that E7 recruited CUL2, driven by CUL2/E2F1/miR-424 regulatory loop, is overexpressed and accelerates HPV16-induced cervical carcinogenesis. Our findings may serve as one of the explanations for a clinical phenomenon that HPV16 possesses the strongest cervical carcinogenicity among high-risk HPV genotypes.

## INTRODUCTION

Cervical cancer is still one of the most common malignancies, ranking as the fourth most common in women worldwide and the second in less developed countries [1] Persistent infection with high-risk human papillomavirus (HR-HPV) is the casual factor for both cervical cancer and its precursor lesions, cervical intraepithelial neoplasia (CIN) [2–4]. Totally 14 high-risk HPV genotypes have been identified, of those, HPV16 is the most frequent and responsible for over 50% of all the diseases [5, 6].

E6 and E7, two primary viral early oncoproteins, play key roles in the initiation and progression of cervical cancer by influencing the pathways related to the cellular transformation [7, 8]. Both E6-P53 and E7-retinoblastoma 1 (pRb) interactions have been well identified as two classic pathways in HR-HPV induced cervical carcinogenesis [9]. However, still some consequent events are not well explained. For instance, HPV16 is a genotype that not only represents the most infection frequency but also possesses the most powerful carcinogenic potential. Just based on those clinical evidences, American Society for Colposcopy and Cervical Pathology and other authoritative institutes recommend that HPV16+/cytology- women can be referred to colposcopy directly [10, 11], although it has been still unclear yet why HPV16 possesses the strongest carcinogenic capacity.

Cullin 2 (CUL2) was initially identified as an essential scaffold protein required for the full activity of RING-genotype E3 ubiquitin ligase complexes (CRLs), the largest class of E3 ligases with more than 200 members [12, 13]. Huh et al [14] recently identified that CUL2 acted as a specific HPV16 E7-associated cellular protein and HPV16 E7-associated CUL2 ubiquitin ligase complex contained pRb, which was regarded to contribute to promoting HPV16 E7-mediated pRb degradation. Furthermore, White et al [15] found that only HPV16 E7 oncoprotein possessed the ability to bind to CUL2, when characterizing the interaction between E7 and cellular proteins in 17 different HPV genotypes. Since HPV E7-mediated pRb destabilization is a universal mechanism among all high-risk HPV genotypes, CUL2 may play a specific role in enhancing the action of E7 of HPV16. Thus, the insight into the function of CUL2 may facilitate to understand the mechanism by which HPV16 possesses the most powerful potential, among all genotypes, to induce cervical carcinogenesis.

Here, we initially observed CUL2 mRNA and protein expression and the correlation with HPV16 E7 expression in HPV16 positive cervical cancer and its precursor tissues, confirmed CUL2 potential role in clinic and function in cell growth *in vitro* and tumorigenicity *in vivo*, and then verified the existence of CUL2/E2F transcription factor 1 (E2F1)/miR-424 regulatory loop and its contribution to CUL2 overexpression in HPV16 positive cancer cells. Our results suggest that CUL2 overexpression driven by CUL2/E2F1/miR-424 regulatory loop accelerates HPV16-induced cervical carcinogenesis.

## RESULTS

### CUL2 is overexpressed and correlated with HPV16 E7 in HPV16 positive cervical cancer and its precursor tissues

**Figure 1:**
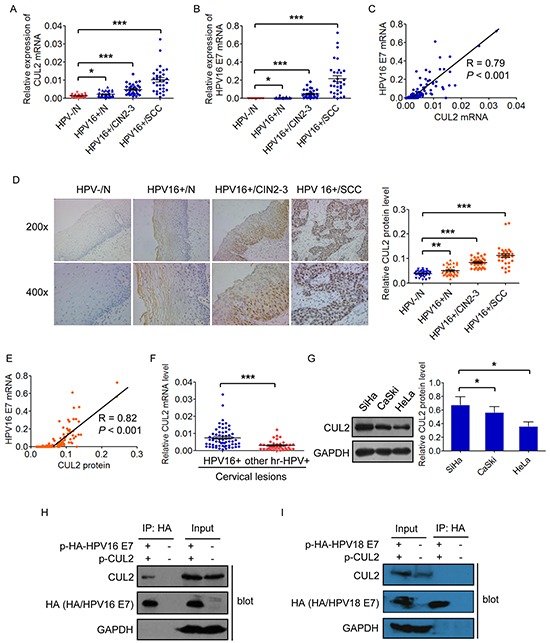
CUL2 is overexpressed and associated with HPV16 E7 in HPV16 induced cervical oncogenesis **A.** and **B.** Assessment of CUL2 and HPV16 E7 mRNA expressions by real time-PCR analysis in total RNAs derived from 137 cervical samples. Data are presented as mean ± SEM. **C.** Correlation between the mRNA expressions of CUL2 and HPV16 E7. **D.** HCC sections were subjected to immunohistochemistry for CUL2 protein. Data are presented as mean ± SEM. **E.** Correlation between the CUL2 protein and HPV16 E7 mRNA levels. **F.** Expression of CUL2 mRNA in two groups of cervical lesion tissues with HPV16 infection or other high risk HPV (hr-HPV) infection. Data are presented as mean ± SEM. **G.** CUL2 expression levels among three cervical cancer cell lines. Data are presented as mean ± SD of three independent experiments. **H.** 293T cells were transfected with HA-tagged HPV16 E7 expression plasmid and CUL2 expression plasmid or equal empty vectors. 24h post-transfection, cell lysates were subjected to immunoprecipitation (IP) with anti-HA antibody, followed by western blot for CUL2, HA-E7, and GAPDH as a control. **I.** 293T cells were transfected with HA-tagged HPV18 E7 expression plasmid and CUL2 expression plasmid or equal empty vectors. 24h post-transfection, cell lysates were subjected to immunoprecipitation (IP) with anti-HA antibody, followed by western blot for CUL2, HA-E7, and GAPDH as a control. *P<0.05; **P<0.01; ***P<0.001.

We initially examined CUL2 and HPV16 E7 mRNA expressions using qRT-PCR in a panel of 137 cervical specimens, including 36 HPV negative and 37 HPV16 positive normal cervical tissues, 33 HPV16 positive CIN 2-3, and 31 HPV16 positive invasive cervical cancer tissues. Gradually increased expressions of CUL2 mRNA was determined from HPV negative normal cervix throughout HPV16 positive normal cervix and HPV16 positive CIN2-3 to HPV16 positive cervical cancer tissues, with an increase of 1.64-fold (*p* = 0.047), 3.77-fold (*p* = 7.73×10^−10^), and 7.98-fold (*p* = 6.75×10^−11^), respectively, compared to HPV negative normal cervical tissues (Figure 1A). Similarly, the expression of HPV16 E7 mRNA was also significantly increased across HPV16 infection progression (Figure 1B). Furthermore, there was a strong positive correlation between the expressions of CUL2 and HPV16 E7, with Spearman's correlation coefficient values (R) at 0.79 (*p* = 1.00×10^−13^) (Figure 1C and Supplementary Table S2).

Similar to mRNA detection, the expression of CUL2 protein by IHC analysis was negative or weak in HPV negative cervical normal epithelium, slightly increased in HPV16 positive cervical normal tissues with the localization in the cytoplasm in the upper layers of epithelium, while greatly increased and located both in nucleus and cytoplasm in the midzone and basal layer of the infected epithelium in HPV16 positive CIN2-3, as well as invasive cancer tissues (Figure 1D). The CUL2 protein level was also obviously correlated with HPV16 E7 mRNA level (R = 0.82, *p* = 1.00×10^−13^) (Figure 1E and Supplementary Table S2). Our findings suggest that CUL2 overexpression may participate in the early stage of HPV16 E7 induced carcinogenesis.

We next examined whether CUL2 expression pattern was associated with HPV genotypes, and found that the expression of CUL2 mRNA in the 64 HPV16 positive cervical lesion tissues was much higher than that in 39 other than type 16 high-risk HPVs positive tissues (Figure 1F). We then chose the three most well-known cervical cancer cell lines, HPV16 positive SiHa and CaSki cell lines and HPV18 positive HeLa cell line, to confirm the clinical observation in *vitro*. As expected, CUL2 expression was higher in SiHa and CaSki cells than that in HeLa cells (Figure 1G). In addition, we co-transfected HA-tagged HPV16 E7 or HPV18 E7 with CUL2 expression plasmid into 293T cells, and found by Co-IP assay that CUL2 was combined with E7 of HPV16 only (Figure 1H), but not HPV18 E7 (Figure 1I). Taken together, our results suggest that CUL2 overexpression is preferably related to HPV16-induced cervical carcinogenesis.

### CUL2 enhances the proliferation and tumorigenicity of cervical cancer cells in vitro and in vivo

**Figure 2:**
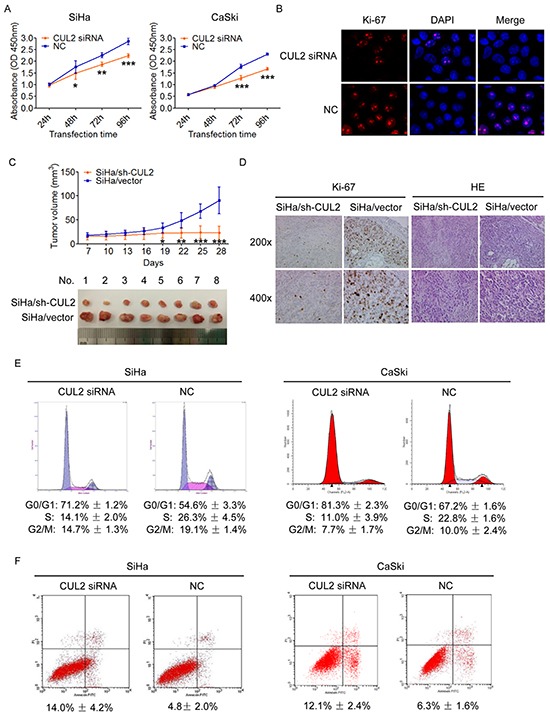
Inhibition of CUL2 suppresses the proliferation, blocks the cell cycle G1-S transition and promotes apoptosis of cervical cancer cells *in vitro* and suppresses the tumorigenicity *in vivo* **A.** SiHa and CaSki cells were harvested at 24h, 48h, 72h, and 96h after transfection, and CCK8 assay were performed. Data show mean ± SD of three independent experiments. **B.** SiHa and CaSki cells were treated with CUL2 siRNA or NC, and analyzed by fluorescence microscopy. Ki-67 and DAPI staining are shown. **C.** Tumor xenograft model. The two group cells were injected into the right-side and left-side axilla of nude mice (n=8), respectively. Data points are presented as mean ± SD tumor volume. **D.** Histopathology of xenograft tumors. The tumor sections were under IHC staining using antibody against Ki-67 and H&E staining. **E.** Flow cytometric analysis of the percentage of SiHa and CaSki cells in various phases of the cell cycle at 48h after transfection with CUL2 siRNA and NC, respectively. Data are representative of three independent experiments. **F.** Flow cytometric analysis of apoptosis in SiHa and CaSki cells at 72h post transfection. Data are presented as mean ± SD of three independent experiments. *P<0.05; **P<0.01; ***P<0.001.

We transfected specific CUL2 siRNA or siRNA negative control into HPV16 positive SiHa and CaSki cells, and examined the effects of CUL2 on cellular proliferation. CCK8 detection showed that transient inhibition of CUL2 evidently reduced the proliferation rate of SiHa and CaSki cells (Figure 2A). Immunofluorescence assay found that inhibition of CUL2 could decrease the expression of Ki-67, a well-known marker of proliferation in SiHa cells (Figure 2B), compared to negative control-transfected cells.

We further engineered SiHa-based cells that expressed short hairpin RNA (shRNA) for CUL2 by lentiviral transduction (Supplementary Figure S1). Those CUL2-suppressing and control cells were subcutaneously injected into the right-side and left-side axilla of 8 nude mouse, respectively, and tumor size was monitored every 3 days for a period of 4 weeks. As shown in Figure 2C, the tumors in the SiHa/sh-CUL2 group showed visibly slower growth than those in the SiHa/vector group. Differences in tumor volumes became more apparent after 19 days of transplantation and persisted until the experimental endpoint, when tumor volumes in controls were average 4.0-fold larger than those injected with SiHa/sh-CUL2 cells (n = 8; *p* = 2.18×10^−5^) (Figure 2C). IHC staining confirmed that xenograft tumor tissues with suppression of CUL2 displayed much lower Ki-67 indexes than the controls (Figure 2D), suggesting that CUL2 acts as an oncogene through its pro-proliferation activity during HPV16 induced cervical carcinogenesis.

### CUL2 accelerates G1-S cell cycle progression and reduces early cell apoptosis

We again found by PI staining that inhibition of CUL2 in SiHa and CaSki cells triggered a significant accumulation of cells in G0/G1 phase and reduced the percentage of cells in S phase (Figure 2E). Moreover, we observed using Annexin V & *P*I apoptosis detection that the rate of early apoptosis was elevated by ~2.9 and ~1.9-fold in SiHa and CaSki cells with CUL2, compared to the control group, respectively (Figure 2F). Our findings indicate that CUL2 promotes cell growth through cell cycle progression and apoptosis.

### CUL2 is a direct target of miR-424, but reversely suppresses miR-424

**Figure 3:**
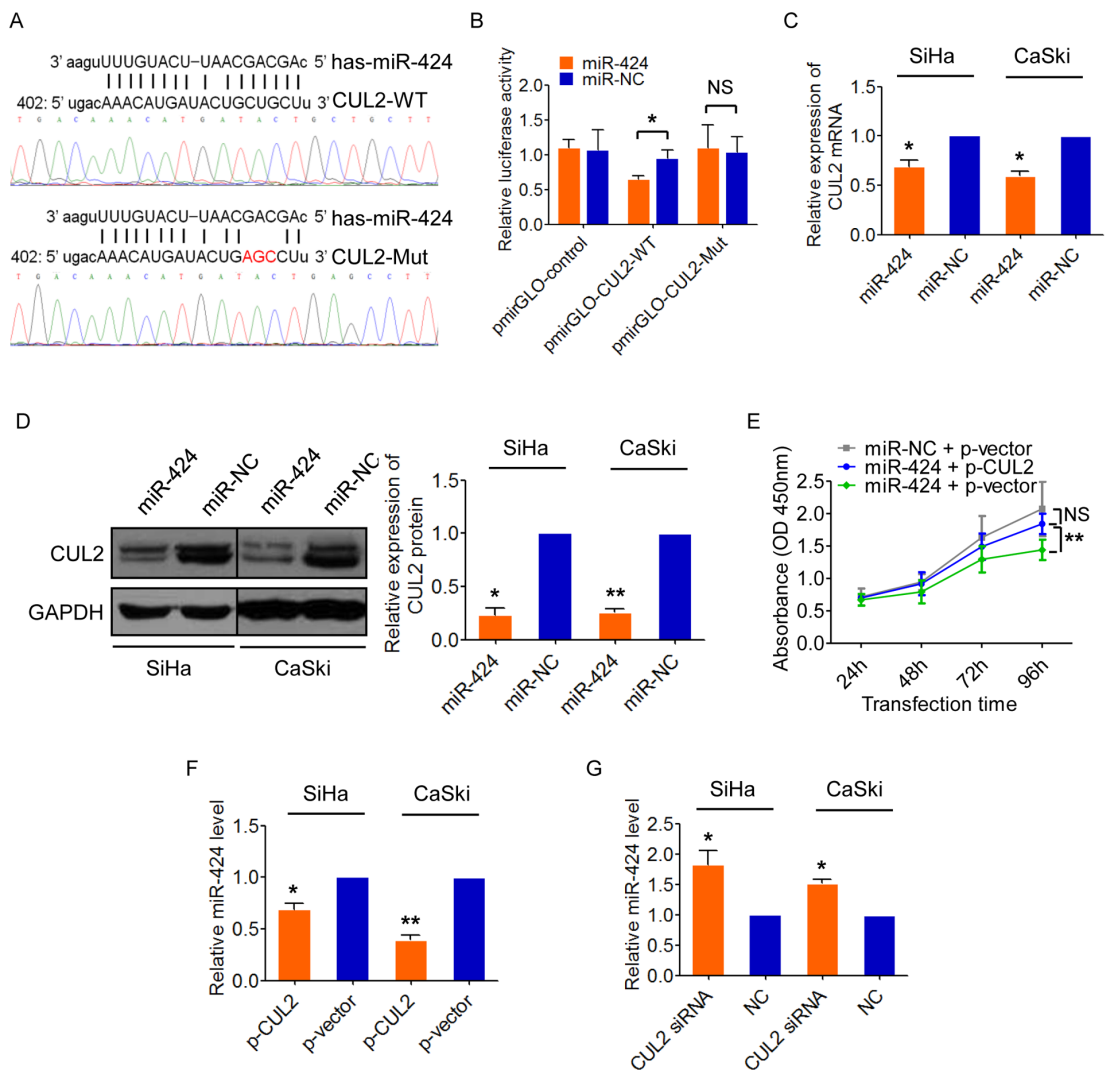
CUL2 is a direct target of miR-424, but also can negatively regulates miR-424 **A.** Predicted target site of miR-424 in the 3′;UTR of CUL2 and reporter constructs, containing a WT or mutated CUL2 3′;UTR. **B.** WT or mutant or empty reporter constructs were co-transfected into SiHa cells with miR-424 mimic or mimic negative control. Luciferase activity was determined. Data represent the average of three independent experiments ± SD. **C.** and **D.** CUL2 mRNA and protein levels in SiHa and CaSki cells transfected with miR-424 or miR-NC. Data are presented as mean ± SD of three independent experiments. **E.** SiHa cells were transfected with miR-424 mimic 12h prior to transfection with CUL2 expression plasmid or empty vector control. SiHa cells transfected with miRNA negative control and empty vector were used as control. Cells were harvested at 24h, 48h, 72h, and 96h after transfection, and CCK8 assay was performed. Data show mean ± SD of three independent experiments. **F.** and **G.** CUL2 negatively regulated miR-424 expression in SiHa and CaSki cells by qRT-PCR. Data are presented as mean ± SD of three independent experiments. *P<0.05; **P<0.01; ***P<0.001; NS, not significant.

While investigating the molecular mechanism of CUL2 overexpression in cervical carcinogenesis, we observed that the 3′;UTR of CUL2 has a putative miR-424 binding site (TargetScan, Figure 3A). Co-transfection of CUL2 3′;UTR luciferase reporter with miR-424 mimic significantly repressed the activity of the reporter construct containing a wild-type but not a mutated miR-424 binding site (Figure 3A–3B). Furthermore, miR-424 upregulation reduced the levels of CUL2 mRNA (Figure 3C) and protein expression (Figure 3D) in both SiHa and CaSki cells. Similar to the action of CUL2 inhibition, miR-424 overexpression reduced cell growth by blocking cell cycle progression and inducing apoptosis in cervical cancer cells, as we previously reported [16]. We then determined whether CUL2 is required for the effects of miR-424. Overexpression of CUL2 abolished the effects of miR-424 on cell proliferation (Figure 3E). These results indicate thatCUL2 is a direct target of miR-424.

Interestingly, miR-424 expression was decreased in SiHa cells after CUL2 overexpression (Figure 3F) and increased after CUL2 silence with siRNA (Figure 3G), suggesting there might be a feedback regulatory loop between CUL2 and miR-424, which consequently results in sustained CUL2 overexpression in HPV16 positive cervical cancer cells.

### E2F1 directly suppresses miR-424 expression

**Figure 4:**
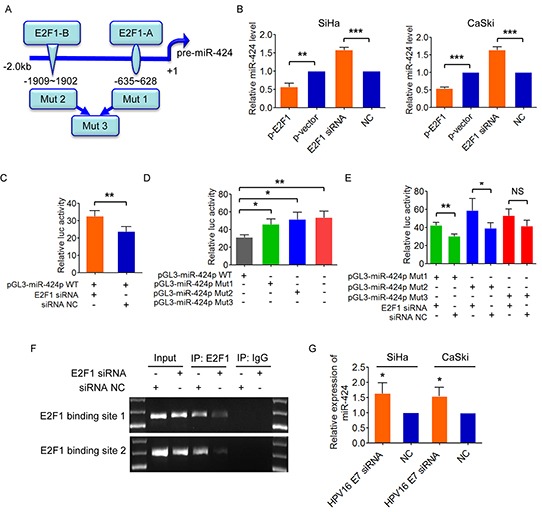
E2F1 negatively regulates miR-424 **A.** Predicted binding sites of E2F1 on *miR-424* regulatory region on chromosome X. Deleting mutation of putative E2F1 binding sites A and B was shown as mut 1 and mut 2, respectively. Deletion of the both two binding sites was shown as mut 3. **B.** SiHa and CaSki cells were transfected with E2F1 expression plasmid or siRNA, and miR-424 expression was measured, respectively. **C.** Luciferase activity of wild-type miR-424 promoter reporter construct after inhibition of E2F1 by siRNA. **D.** Identification of E2F1 interacting region by using luciferase reporter-containing wild-type, mut1, mut2 and mut3 promoter region of pre-miR-424. **E.** Luciferase activity of miR-424 promoter-mut1, -mut 2, and -mut3 after co-transfected with E2F1 siRNA or control. **F.** ChIP assays were performed. Total inputs are indicated. All quantitative data are represented as mean ± SD of three independent experiments. **G.** SiHa and CaSki cells were transfected with HPV16 E7 siRNA or NC, and analyzed by qRT-PCR. miR-424 expression is shown. Data show mean ± SD of three independent experiments. *P<0.05; **P<0.01; ***P<0.001; NS, not significant.

Next, we examined potential common transcription factor binding sites in miR-424 promoter areas and found two moderately conserved E2F1 motifs (referred to as A and B) in miR-424 promoter regions (Figure 4A). We then upregulated E2F1 expression by plasmid or silenced E2F1 by siRNA in SiHa and CaSki cells, and observed that miR-424 expression level was decreased or increased in response to the changed E2F1 expression (Figure 4B), suggesting that E2F1 is a repressor of miR-424. Furthermore, we tested the activity of miR-424 upstream regulatory region by cloning a 2.0 kb fragment spanning the two E2F1 binding sites into pGL3-Basic luciferase reporter plasmid. This activity was stimulated by E2F1 knockdown (Figure 4C). Site A and B were then mutated by deletion and compared to its wild-genotype counterpart. Either loss of the E2F1 binding site A or B or both sites led to higher reporter activity, compared to the wild-genotype reporter construct (Figure 4D). In addition, the luciferase activity was still enhanced with E2F1 knockdown when the reporter construct carried only one mutant of the two binding sites (A or B), but not changed when the reporter construct carried the deleted mutants of both binding sites (Figure 4E), indicating that the two binding sites are required for E2F1 mediated suppression of miR-424. Furthermore, E2F1-Chromatin immunoprecipitation (ChIP) specificity also showed that E2F1 suppression by E2F1 siRNA resulted in reduced E2F1 occupation on the miR-424 promoter (Figure 4F) Thus, our results suggest that E2F1 functions as a transcriptional repressor of miR-424 in cervical cancer cells.

Considering E2F1 expression is promoted by E7, we examined the effect of E7 on miR-424, a downstream as demonstrated as above. As we expected, inhibition ofHPV16 E7 suppressed the expression of miR-424 in both SiHa and CaSki cells (Figure 4G).

### CUL2 is associated with E2F1 and promotes E2F1 expression

**Figure 5:**
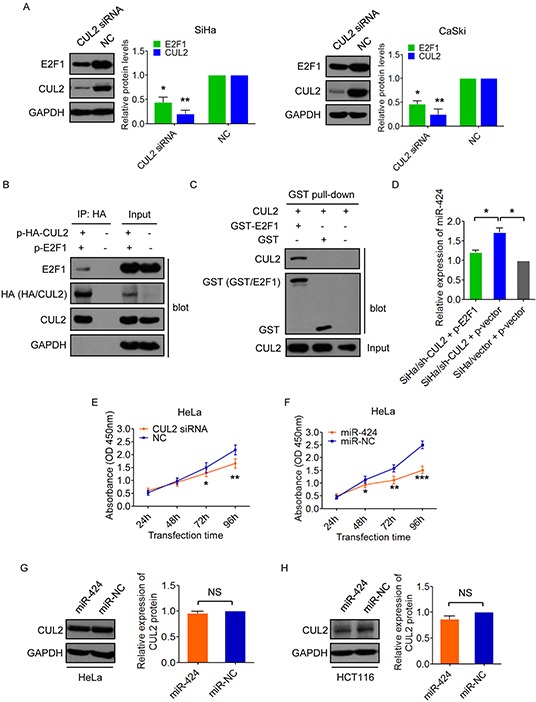
CUL2 binds and regulates E2F1 **A.** CUL2 positively regulated E2F1 protein expression in SiHa and CaSki cells. Data represent the average of three independent experiments ± SD. **B.** 293T cells were cotransfected with HA-tagged CUL2 expression plasmid and E2F1 expression plasmid, or equal empty vectors. 24h post-transfection, cell lysates were subjected to immunoprecipitation (IP) with anti-HA antibody, followed by western blot for E2F1, HA, CUL2, and GAPDH as a control. **C.** E2F1 bound to CUL2 *in vitro*. Bacterially produced GST-E2F1 fusion protein or GST was incubated with 293T cell lysates and analysis by GST pull-down kit, followed by western blot for CUL2 and GST. Inputs are also indicated. **D.** SiHa/sh-CUL2 cells were transfected with p-E2F1 and p-vector, respectively. miR-424 expression level was analyzed by qRT-PCR. SiHa/vector cells were used as control. Data show mean ± SD of three independent experiments. **E.** HeLa cells were treated with CUL2 siRNA or NC, and cell proliferation was analyzed by CCK-8 assay. Data show mean ± SD of three independent experiments. **F.** HeLa cells were harvested at 24h, 48h, 72h, and 96h after transfection with miR-424 mimic or miR-NC, and CCK8 assay were performed. Data show mean ± SD of three independent experiments. **G.** and **H.** CUL2 protein levels in HeLa and HCT116 cells transfected with miR-424 or miR-NC. Data are presented as mean ± SD of three independent experiments. *P<0.05; **P<0.01; ***P<0.001; NS, not significant.

As E2F1 directly repressed miR-424 expression, we tested the possibility of CUL2 mediating E2F1-regulated miR-424 inhibition in SiHa and CaSki cells. own regulation of CUL2 expression decreased the expression of E2F1 protein in both SiHa and CaSki cells (Figure 5A). Interestingly, E2F1 was found to be associated with CUL2 by Co-IP assay (Figure 5B). We further set up *in vitro* GST pull-down assay to identify interaction between CUL2 and E2F1. As shown in Figure 5C, GST-E2F1 bound to CUL2, whereas GST alone did not bind to CUL2. Thus, the results suggest that CUL2 binds to E2F1 and promotes E2F1 expression.

To further determine whether E2F1 is critical for CUL2 mediated miR-424 suppression, we overexpressed E2F1 in CUL2-suppressing SiHa cells (SiHa/sh-CUL2) and evaluated miR-424 expression level. E2F1 addition ablated the increase in the level of miR-424 in SiHa/sh-CUL2 cells to levels similar to control SiHa/vector cells (Figure 5D), indicating that E2F1 is required for CUL2 mediated miR-424 inhibition. Also, we tested the functional effects of E2F1 on growth of SiHa cells, and found that E2F1 exerts similar biological actions to CUL2 (Supplementary Figure S2A–S2D), which was opposite to miR-424.

Taken our results together, we delineate a feedback regulatory loop involving CUL2, E2F1, and miR-424 in HPV16 positive cervical cancer cells, where overexpressed CUL2 promotes E2F1 expression, elevated expressed E2F1 suppresses miR-424 in turn, reduced expressed miR-424 thus abates the inhibition to CUL2, and consequently results in persistent CUL2 overexpression.

### The existence of CUL2/E2F1/miR-424 feedback loop is specific to HPV16 genotype

Just as we described above, there was a positive correlation between CUL2 and HPV16 E7 expression in HPV16 positive cervical tissues, CUL2 overexpression existed specifically in HPV16 positive cells and tissues, and CUL2 was only combined with HPV16 E7. To further verify the change of CUL2 is HPV 16 genotype specific, and we examined the function of CUL2 and miR-424 in HPV18 positive HeLa cells, and found that the inhibition of CUL2 could slightly suppress cell growth, while miR-424 obviously suppressed cell growth in HeLa cells (Figure 5E and 5F). The effect of CUL2 on cell growth in HPV18 positive cells was much less than what we observed in HPV16 positive cells. Then, we tested miR-424 regulation to CUL2 in HeLa and HPV negative HCT116 cells. As expected, we did not find that the changes of CUL2 expression in response to miR-424 overexpression in both HeLa (Figure 5G) and HCT116 cells (Figure 5H). Thus, our results suggest that CUL2 overexpression driven by CUL2/E2F1/miR-424 regulatory loop exists specifically in HPV16 infected cervical cancer cells.

### CUL2 expression is correlated with miR-424 and E2F1 expression in HPV16 positive cervical cancer and precursor tissues

**Figure 6:**
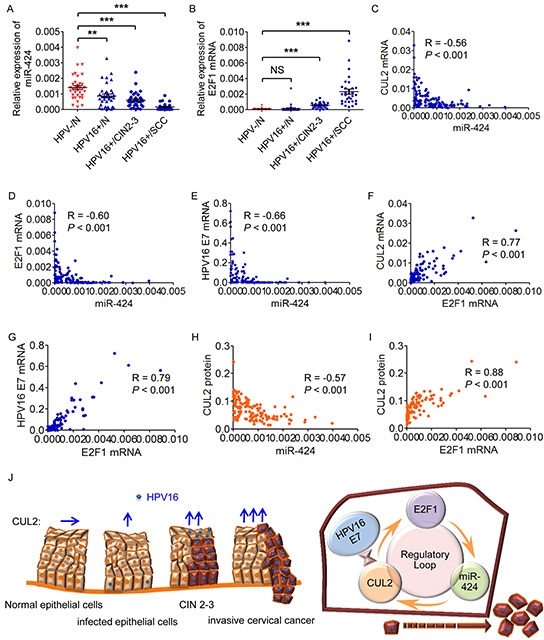
The members of CUL2 regulatory loop are perturbed in HPV16-induced human cervical carcinogenesis **A.** and **B.** Assessment of miR-424 and E2F1 mRNA levels by real time-PCR analysis in total RNA derived from the same 137 cervical tissues. Data are shown as mean ± SD. **P<0.01; ***P<0.001; NS, not significant. **C, D.** and **E.** Correlation between miR-424 and CUL2 or E2F1 or HPV16 E7 mRNA levels. **F.** and **G.** Correlation between the mRNA expression levels of E2F1 and CUL2 or HPV16 E7. **H.** and **I.** Correlation between CUL2 protein levels and miR-424 or E2F1 mRNAs. **J.** The proposed model for the role of CUL2 overexpression driven by CUL2/E2F1/miR-424 regulatory loop during HPV16 induced cervical carcinogenesis. HPV16 E7-CUL2 interaction recruits CUL2 and the activation of regulatory loop among CUL2, E2F1, and miR-424 produces the persistent CUL2 overexpression, and consequently promotes the development of HPV16 induced cervical cancer.

Contrarily to the pattern of CUL2 and HPV16 E7 expression, miR-424 expression was decreased during the stepwise progression from cervical normal epithelium throughout precancer lesions to cervical cancer with HPV16 infection (Figure 6A). We also found that E2F1 mRNA level was increased in CIN 2-3 and cervical cancer tissues compared to normal tissues (Figure 6B), though there was no significant difference of E2F1 expression between normal cervix with and without HPV infection, indicating that aberrant E2F1 level might occur after the morphology change. Furthermore, in the same 137 tissue samples, we tested whether any correlation existed between the expression levels of the different members of the loop and found an inverse correlation between miR-424 and CUL2 or E2F1 or HPV16 E7 mRNA levels (Figure 6C, 6D, and 6E), and a positive correlation between E2F1 and CUL2 or HPV16 E7 mRNA levels (Figures 6F and 6G). Also, miR-424 was negatively associated with CUL2 protein expression, whereas E2F1 was positively associated with CUL2 protein level (Figure 6H and 6I). The correlation information is listed in Supplementary Table S2. As summarized in Figure 6J, our data strongly suggested that the CUL2-E2F1-miR-424 feedback loop is important for the development of HPV16 induced human cervical cancer.

## DISCUSSION

HR-HPVs exist in more than 99% of cervical cancer tissues[17], among them, HPV16 is a predominant genotype and possesses the most powerful carcinogenicity, which is particularly of clinical significance [18]. Viral oncoprotein E7 plays an essential role as same as E6 in the development of cervical cancer. Recent studies revealed that CUL2 participated in the E7-mediated pRb degradation in HPV16, but not other genotypes [14, 15, 19], infected cervical cancer cells. Thus, in HPV16-induced cervical cancer, CUL2 may play a different role from other high-risk HPVs. Considering a stronger carcinogenicity of HPV16 than that of others, it should be valuable to explore the expression, associated regulating mechanism, and carcinogenic potential of CUL2 itself in HPV16-induced cervical cancer.

CUL2 is a member of cullins, a family of molecular scaffolds that organize the largest class of RING E3 ligases (also known as the cullin-RING ligase complexes, CRLs) and required for the ubiquitin-dependent protein degradation [20]. Although other cullin family proteins display their indispensable roles in cell-cycle control, DNA replication, and embryogenesis in model organisms [21–25], the role of CUL2 has not been well studied. It has been shown that CUL2 is an essential regulating gene for mitotic germline proliferation, meiosis, and polarity in *C.elegans* [26–29]. Again in the circumstance of virus infection, CUL2 functions mainly as a scaffold protein of E3 ubiquitin ligase [30, 31]. Here, we showed that CUL2 expression was obviously higher in HPV16 positive cervical cancer cells and tissues that that in other positive high-risk genotypes. Moreover, CUL2 was gradually increased from normal cervix without HPV infection throughout precancer lesions to cervical cancer tissues with HPV16 infection, and positively correlated with HPV16 E7 expression. IHC analysis additionally demonstrated a translocating staining of CUL2 protein from cytoplasm towards nucleus following cervical lesion progress. Such morphology indicates that CUL2 overexpression specifically exists in HPV16 positive cervical cancer cells and tissues and implies that CUL2 may play a more crucial role in the development of HPV16-induced cervical cancer other than participating in E7-mediated pRb degradation. To confirm CUL2 function in HPV16 induced carcinogenesis, we observed the influence of CUL2 on tumorigenicity and growth of cervical cancer *in vitro* and *in vivo* by loss-of-function experiments, and found that enforced inhibition of CUL2 expression evidently suppressed cell proliferation through retarding G1-S transition of cell cycle and inducing early apoptosis in cervical cancer cells. Mouse model also revealed a slowed growth of tumor transplanted by cells with CUL2 knockdown. Our findings suggest that CUL2 may play the direct oncogenic role in the development of HPV16-induced cervical cancer.

To date, the regulator of CUL2 is largely unclear. Recently, we and others have described the existence of dynamic miRNA-target networks in a variety of cancers [16, 32–34]. We here identified that miR-424 is a direct upstream regulator of CUL2. Interestingly, we also found that CUL2 reversely acted on miR-424. Thus, we supposed there might be a feedback regulatory loop between miR-424 and CUL2 in HPV16 positive cervical cancer cells. Previous studies revealed that some transcription factors (TFs) could control miRNA expression and participate in various biological processes, including oncogenesis [35–38]. For example, E2F1-regulated miRNAs function as tumor suppressors to arrest cell-cycle and induce apoptosis [39, 40], and E2F1-miR-223 negative feedback loop plays a role in acute myeloid leukemia [41]. Thus, it is possible that CUL2 regulates miR-424 through E2F1. We found that HPV16 E7 suppressed miR-424 expression (Supplementary Figure S3). Also, it has been known that HPV16 E7 activates E2F1-driven transcription [42], and E2F1 is a master regulator for promoting G1 to S-phase transition [43] and an essential signal in apoptosis [44], implying that E2F1 may serve as a candidate transcription factor. As expected, we found that two E2F1 binding sites located ~2 kb upstream from coding sequence of miR-424 on chromosome X. We then confirmed that E2F1 suppressed miR-424 expression through directly binding to the two functional binding sites of miR-424 promoter regions. We further verified that CUL2 bound to E2F1 and promoted E2F1 expression, and E2F1 was required for CUL2 mediated miR-424 inhibition in cervical cancer cells. Though other mechanisms may also affect E2F1 expression and further systemic experiments should be done to address this issue, our results verified the existence of a definite regulatory loop among CUL2, E2F1, and miR-424, which results in the persistent overexpression of CUL2, in HPV16 positive cervical cancer cells.

In summary, we identify, for the first time to our knowledge, that E7 recruited CUL2 plays a key role in cervical carcinogenesis induced by HPV16. HPV16 E7-CUL2 interaction recruits CUL2 and the activation of regulatory loop among CUL2, E2F1, and miR-424 produces the persistent CUL2 overexpression, which consequently promotes the growth of cervical cancer cells. Our findings may serve as one of the explanations for the most powerful cervical carcinogenicity of HPV16 among high-risk HPV genotypes.

## MATERIALS AND METHODS

### Collection of clinical specimens

Human cervical biopsied tissue specimens and exfoliated cells including disease-free histologically normal cervical specimens (n=36), specific HPV16-infected normal cervix (n=37), specific HPV16-infected CIN 2-3 (n=33), and specific HPV16-infected cervical cancer (n=31) were collected from Women's Hospital, School of Medicine, Zhejiang University. All procedures involving human cervical tissues were approved by the Women's Hospital Research Ethical Committee of Zhejiang University and the specimens were obtained with written informed consent. Further details are provided in the Supplementary Methods.

### Total RNA isolation and quantitative real-time PCR

RNA extraction was performed as described previously [16]. Primers of E2F1 were purchased from Bioneer, and other sequences of oligonucleotides used as qPCR primers are listed in Supplementary Table S1. Further details are provided in the Supplementary Methods.

### Cell culture and transfection

HPV16-positive human cervical epithelial carcinoma SiHa and CaSki cells and HPV18-positive cervical cancer HeLa cells (purchased from the ATCC) were all grown in Dulbecco's modified Eagle's medium (DMEM, Gibco) supplemented with 10% fetal bovine serum (FBS, Gibco). Human embryonic kidney cell line HEK293T (obtained from the ATCC) was maintained in RPMI-1640 (Gibco) with 10% FBS. The human colorectal cancer cell line HCT116 was cultured in McCoy's 5A complete medium (Gibco) with 10% FBS. All incubations were carried out in humidified atmosphere of 5% CO_2_ at 37°C.

CUL2, E2F1, HPV16 E7, HPV18 E7 CDS were subcloned to pcDNA3.1+ vector using BamH1/ XhoI sites. Specific siRNA targeting CUL2 and negative control siRNA (NC) were purchased from GenePharma, Shinghai, China. siRNA targeting E2F1 and negative control siRNA were purchased from Bioneer. miR-424 mimic and nonspecific miRNA negative control were purchased from Dharmacon, Lafayette, CO, USA.

Plasmids were transfected using X-tremeGENE HP DNA transfection reagent (Roche). siRNA and miRNA mimic were transfected using DharmaFECT1 transfection reagent (Dharmacon) as per manufacturer's protocol.

### Cell proliferation analysis

SiHa, CaSki, and HeLa cells were plated in 96-well plates in triplicate at 5×10^3^ cells/well, transfected with siRNA, and then cultured for 24h, 48h, 72h, and 96h. The absorbance at 450nm was measured after incubation with 10 μl of CCK8 (Dojindo) for 1h.

### Lentiviral transduction and animal model experiments

Details of lentiviral transduction are provided in the Supplementary Methods. For *in vivo* tumor growth assay, 4 week-old BALB/c nude mice were obtained from Shanghai Laboratory Animal Center (SLAC Shanghai, China) and maintained in the animal facilities of the Laboratory Animal Center of Zhejiang University. All animal protocols were in accordance with the institutional guidelines and regulations of the use and care of laboratory animals and approved by the Animal Care Committee of Zhejiang University. Briefly, equal numbers of SiHa/sh-CUL2 or SiHa/vector (1.2×10^7^ cells/injection) were resuspended in 150μl PBS, and injected subcutaneously into the right-side or left-side axilla of each mouse (n=8). The diameters of tumors were measured every 3 days with precision calipers. Tumor volume based on caliper measurements were calculated by the formula: volume = [(tumor length × tumor width) ^2^] / 2. 4 weeks after xenograft, mice were sacrificed, and tumors were removed and photographed. The tissue samples were fixed in 10% paraformaldehyde, and embedded in paraffin for histological evaluation.

### Luciferase reporter assay

Detailed luciferase reporter assay for miR-424 directly targeting CUL2 3′;UTR are provided in the Supplementary Methods. In case of promoter assay, miR-424 promoter-Luc-wt reporter plasmid, a 2.0 kb fragment containing two E2F1 binding sites was synthesized by GeneScript and cloned into Xho I and Hind III sites of pGL3-Basic vector (Promega). For the construction of miR-424 promoter-Luc mutant plasmid, the E2F1 binding site A, B and AB were deleted from the miR-424 promoter-Luc-wt construct using QuikChange XL-mutagenesis (Stratagene), respectively. SiHa cells were transfected with these reporter constucts or cotransfected E2F1siRNA or siRNA control with these reporter constucts. A Renilla luciferase plasmid (PRL-TK from Promega) was cotranfected as an internal control. Cells were harvested 24h after transfection, and the luciferase activities of the cell lysates were measured by Dual-luciferase reporter assay.

### Chromatin immunoprecipitation

ChIP assays were performed using the EZ-Zyme™ Chromatin Prep Kit and EZ-Magna ChIP™ G Chromatin Immunoprecipitation kits (Millipore) according to the manufacturer's standard protocol. ChIP-DNA was quantified using RT-PCR and the enrichment was expressed as fold enrichment compared with IgG. The antibody used was: anti-E2F1 (Millipore). The primers specific for E2F1 binding sites listed in Supplementary Table S1.

### Immunoprecipitation (Co-IP assay)

293T cells were transfected with various plasmids as required and harvested at 24h post-transfection. Cell extracts were prepared in a modified Pierce IP Lysis Buffer containing Halt Protease Inhibitor Single-Use Cocktail (Thermo) by 5 minutes incubation on ice. Samples were centrifuged at 13,000 g for 10 minutes at 4°C, and supernatants were immunoprecipitated with anti-HA agarose beads at 4°C overnight with end-over-end minxing. The immunoprecipitation was performed using Pierce HA Tag IP/Co-IP Kit (Thermo) following the manufacturer's standard protocol. Immunoprecipitated samples were then solubilized in 2× Non-Reducing sample buffer containing DTT, boiled and separated by SDS-PAGE, and analyzed by western blot.

### GST pull down assay

GST and recombinant GST-tagged E2F1 (pGEX-4T-1-E2F1) were expressed and purified from BL21 strain of *Escherichia coli,* and label as “bait lysate”. GST pull down experiment was performed using Pierce GST Protein Interaction Pull-Down Kit (Thermo) according to the manufacturer's recommended protocol. Equilibrate the Glutathione Agarose resin, add prepared GST and GST-tagged fusion protein (bait), respectively, and incubate at 4°C for 2h with gentle rocking motion to immobilize bait protein. 293T cells were transfected with CUL2 expression plasmid and lysates were prepared 24h post-transfection in Pull-Down lysis buffer containing protease inhibitor cocktail, labeling as “prey lysate”. Approximately 200 μg of prey protein was used per reaction and mixed with the immobilized GST or GST-tagged bait protein. Reaction mixtures were incubated at 4°C for 4h and then washed with Elution buffer. The bound preteins were analyzed by SDS-PAGE.

### Statistical analysis

Statistical calculations were performed using SPSS 21.0 (SPSS, Inc., USA) and GraphPad Prism 5 (GraphPad software, Inc., USA). Results were presented as mean ± SE/SD. Gene or miRNA expression between different groups of cervical tissues were assessed by the nonparametric Mann-Whitney *U* test, and correlations were then analyzed by Spearman rank correlation. Statistical significance of tumor growth was analyzed using a paired Student's t test. All *in vitro* experiments were performed at least in triplicate, and evaluated using student's t test. Data are represented as 2^−ΔCT^ for tissue validation and 2^−ΔΔCT^ for cell line experiments. All *p* values were two-sides and *p* values < 0.05 were regarded as statistically significant. *P<0.05; **P<0.01; ***P<0.001; NS, not significant.

Please refer to Supplementary Data for complete details of Methods.

## SUPPLEMENTARY MATERIALS FIGURES AND TABLES


