# Supplementary material for: CUL2 overexpression driven by CUL2/E2F1/miR-424 regulatory loop promotes HPV16 E7 induced cervical carcinogenesis
Source: Oncotarget. 2016 May 2;7(21):31520–33. doi: 10.18632/oncotarget.9127 (PMC5058775; doi:10.18632/oncotarget.9127)
Supplement: Supplementary file 1 [file oncotarget-07-31520-s001.pdf]

CUL2 overexpression driven by CUL2/E2F1/miR-424 regulatory loop promotes HPV16 E7 induced cervical carcinogenesis

SUPPLEMENTARY METHODS

Clinical sample collection and evaluation of HPV status in clinical samples

Briefly, patients with abnormal cytology and HR-HPV infection underwent a colposcopy-directed biopsy. The exfoliated cells were obtained after HPV testing (HC II) test or thinprep cytological test. Cervical normal tissues without HPV infection were collected from patients who underwent hysterectomy for benign gynecological disease. All samples were immediately snap-frozen in liquid nitrogen and stored at -80°C until use.

Genomic DNA was extracted using DNA purification kit (Qiagen) according to the manufacturer's standard protocol. Flow-through hybridization (HybriMax) was used for HPV genotyping. Normal tissue samples without HPV infection were precisely determined by PCR using L1 consensus primers.

Total RNA isolation and quantitative real-time PCR

RNA was extracted from primary cervical tissues or cultured cells using Trizol reagent (Invitrogen) according to the manufacturer's instructions. For mRNA and miRNA analysis, cDNA was generated from 2μg total RNA per sample using the PrimeScript RT reagent Kit (TaKaRa). Quantitative real-time PCR (qRT–PCR) was performed using SYBR Premix Ex Taq (TaKaRa). mRNA and miRNA expression were normalized using detection of *EEF1A1* and *U6*, respectively. Data are represented as $2^{-\Delta CT}$.

Immunohistochemical staining and analysis

Anti-CUL2 antibody (Abcam, ab1870) was used for immunohistochemistry (IHC) staining of paraffin-embedded cervical tissues. Anti-Ki-67 antibody (Dako, IR626) was used for IHC staining of the xenograft tumors. IHC was carried out as previously described (53). Stained section of each slide was taken pictures on a microscope by an observer with no prior knowledge of the pathological results, and IHC scoring analysis was evaluated by Image pro-plus 6.0 (Media Cybernetics) according to the manufacturer's instructions. The output for the algorithm was mean density (mean density = ((IOD (integrated optical density) SUM) / Area). The average score for each slide was used for statistical analysis.

Western blot analysis

Proteins were electrophoresed on an SDS 8% or 12%-acrylamide gel and transferred to PVDF membrane (Millipore). After blocked with 5% nonfat milk, the membrane was incubated with rabbit anti-CUL2 (1:1000, Abcam, ab1870), rabbit anti-E2F1 (1:2000, Abcam, ab179445), or mouse anti-GAPDH antibody (1:2000, Santa Cruz, sc-47724). A horseradish peroxidase-conjugated secondary antibody (anti-mouse or anti-rabbit, 1:1000, Dawen Biotec) was used for detection. Antibody binding was detected using the enhanced chemiluminescent kit (Biological Industries).

Immunofluorescence

Cells cultivated on glass cover slides were fixed in 4% paraformaldehyde (Sigma) for 20 minutes, permeabilized in 0.1% Triton X-100 (Sigma) for 10 minutes, and blocked in 4% BSA (Sigma) for 1 hour. Proteins were detected with anti-Ki-67 antibody (1400, CST, 9449). Chromatin was stained by DAPI (Vector laboratories). Images were acquired with fluorescence microscopy (Olympus).

Lentiviral transduction

Lentiviral short hairpin RNA (shRNA) clone targeting CUL2 was purchased from Genechem, China. EGFP and scramble shRNA were used as controls. The highest infection efficiency was obtained at MOI of 20 for SiHa cells and confirmed by western blot (Supplementary Figure S1A and S1B). Approximately 1×10^9virus particles were used to transduce SiHa cells for further study.

Cell cycle analysis

SiHa and CaSki cells were transfected with siRNA described as above. At 48h after transfection, the adhered cells were collected, washed with PBS, and fixed with 70% ethanol for 24h at 4°C. A mixture containing propidium iodide (50ug/ml, Sigma), RNase A (100ug/ml, Sigma) and triton X-100(0.2%) was then added to the cells and samples were analyzed 1h after staining with the use of FACS EPICS (Beckman Coulter).

Apoptosis assay

Dual staining with FITC-conjugated Annexin V and PI (Biouniquer) was carried out to detect the induction of apoptotic cell death. Cells were washed with PBS, resuspended in binding buffer containing 3 μl Annexin V-FITC and 3 μl PI. Following incubation for 15 min, cells were analyzed by flow cytometry, and the percentages of Annexin V-positive cells in PI-negative were analyzed.

Luciferase reporter assay

The 3'UTRs of the human CUL2 gene was PCR-amplified (primers are listed in Supplementary Table S1), and cloned downstream of the firefly luciferase gene in pmirGLO vector (Promega), giving rise to the p3'UTR-CUL2 plasmid. These constructs were used to generate the p3'UTRmut-CUL2 plasmid (primers are listed in Supplementary Table S1). SiHa cells were cotransfected 50nm miR-424 mimic or mimic negativel control with 0.1 μg p3'UTR-CUL2 or p3'UTRmut-CUL2. Cells were harvested 24h after transfection and assayed with Dual-luciferase reporter assay system (Promega) according to the manufacturer's instructions. Three independent experiments were performed in triplicate.

SUPPLEMENTARY FIGURES AND TABLES

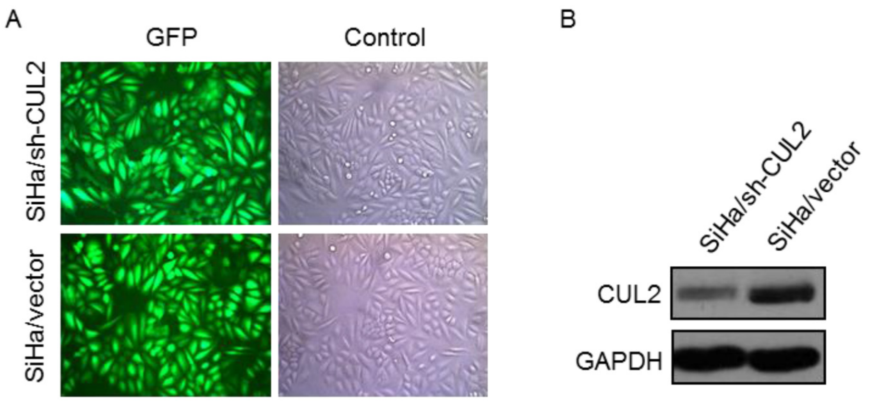

Supplementary Figure S1: Transfection efficiency and expression level of CUL2 inhibition under lentiviral transduction. **A.** and **B.** Infection efficiency in SiHa cells expressing shRNA for CUL2 or vector control was viewed by a green fluorescent protein gene, GFP, and further determined by western blot.

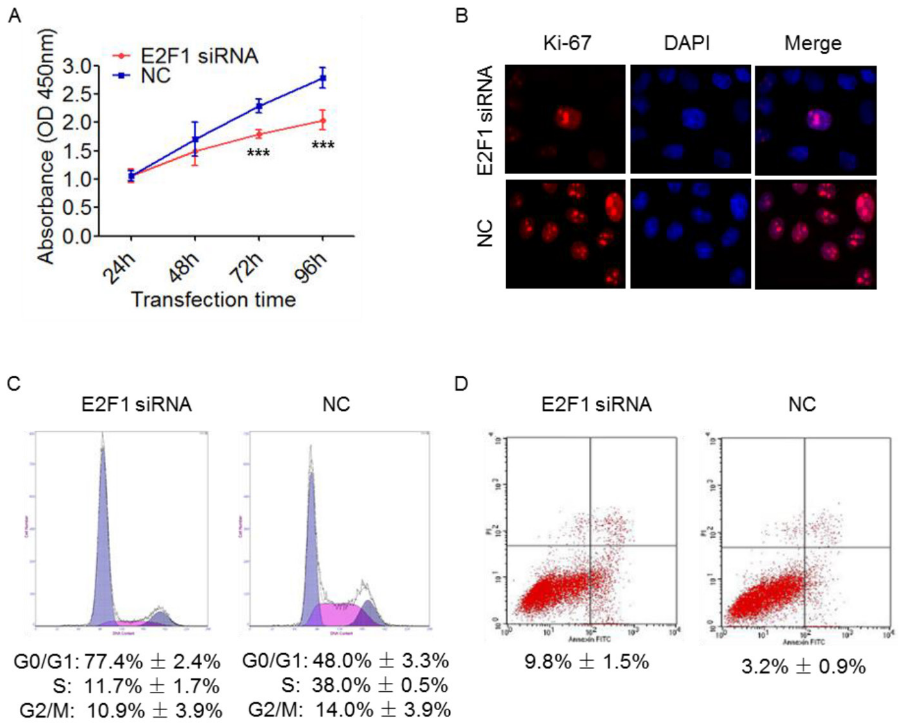

Supplementary Figure S2: Inhibition of E2F1 suppresses the proliferation of cervical cancer SiHa cells by blocking cell cycle progression and promoting apoptosis. A. SiHa cells were harvested at 24h, 48h, 72h, and 96h after transfection, and CCK8 assay were performed. Data show mean ± SD of three independent experiments. ****P*<0.001. **B.** SiHa cells were transfected with E2F1 siRNA or NC, and analyzed by fluorescence microscopy. Ki-67 and DAPI staining are shown. **C.** Flow cyctometric analysis of the percentage of cells in different phases of the cell cycle at 48h after transfection with E2F1 siRNA or NC. **D.** Flow cytometry analysis of apoptosis in SiHa cells at 72h post transfection. All data show mean ± SD of three independent experiments.

Supplementary Table S1: Sequences of Primers

Primers	Sequence (5’ - 3’)
EEF1A1 forward	TGCGGTGGGTGTCATCAAA
EEF1A1 reverse	AAGAGTGGGGTGGCAGGTATTG
CUL2 forward	TATGGCTATGGTGGTGTA
CUL2 reverse	ACTTTCTGGTTTGGGTCT
U6 RT	AACGCTTCACGAATTTGCGT
U6 forward	CTCGCTTCGGCAGCACA
U6 reverse	AACGCTTCACGAATTTGCGT
miR-424 RT	GTCGTATCCAGTGCAGGGTCCGAGGTATTCGCACTGGATACGACTTCAAA
miR-424 forward	CGAAGCAGCAGCAATTCATG
miR-424 reverse	GTGCAGGGTCCGAGGT
HPV16 E7 forward	CATGGAGATACACCTACATTGC
HPV16 E7 reverse	CACAACCGAAGCGTAGAGTC
CUL2 3’UTR forward	CGAGCTCGGAAATTCGGTTGGGTAC
CUL2 3’UTR reverse	GCTCTAGACTGCGATGTCTGTGGAG
CUL2 3’UTR-mut forward	TGACAAACATGATACTGAGCCTTGTCAAATAAAAAAAAAA
CUL2 3’UTR-mut reverse	TTTTTTTTTTATTTGACAAGGCTCAGTATCATGTTTGTCA
E2F1 binding site 1 primer forward	GGCGTATTCTTTGGCTCTT
E2F1 binding site 1 primer reverse	CACCCTAACCCCTTTCATC
E2F1 binding site 2 primer forward	AGTTTCTGTGGAGCTGCTTG
E2F1 binding site 2 primer reverse	GCTGATTCTTACACTATCGTTGC

EEF1A1, eukaryotic translation elongation factor 1 alpha 1; RT, reverse transcriptase; UTR: untranslated region.

Supplementary Table S2: Correlations in the 137 cases of cervical tissues

			CUL2 mRNA	HPV16 E7 mRNA	miR-424	E2F1 mRNA	CUL2 protein
Spearman's rho	CUL2 mRNA	Correlation Coefficient	1.000	.791**	-.556**	.767**	.989**
		Sig. (2-tailed)		0.000	0.000	0.000	0.000
		N	137	137	137	137	137
	HPV16 E7 mRNA	Correlation Coefficient	.791**	1.000	-.661**	.790**	.821**
		Sig. (2-tailed)	0.000		0.000	0.000	0.000
		N	137	137	137	137	137
	miR-424	Correlation Coefficient	-.556**	-.661**	1.000	-.600**	-.565**
		Sig. (2-tailed)	0.000	0.000		0.000	0.000
		N	137	137	137	137	137
	E2F1 mRNA	Correlation Coefficient	.767**	.790**	-.600**	1.000	.800**
		Sig. (2-tailed)	0.000	0.000	0.000		0.000
		N	137	137	137	137	137
	CUL2 protein	Correlation Coefficient	.989**	.821**	-.565**	.800**	1.000
		Sig. (2-tailed)	0.000	0.000	0.000	0.000	
		N	137	137	137	137	137

** Correlation is significant at the 0.01 level (2-tailed).
